# Assessing antimicrobial misuse in small-scale chicken farms in Vietnam from an observational study

**DOI:** 10.1186/s12917-019-1947-0

**Published:** 2019-06-20

**Authors:** Marc Choisy, Nguyen Van Cuong, Truong Dinh Bao, Bach Tuan Kiet, Bo Ve Hien, Ho Viet Thu, Niwat Chansiripornchai, Erry Setyawan, Guy Thwaites, Jonathan Rushton, Juan Carrique-Mas

**Affiliations:** 1 Oxford University Clinical Research Unit, Hanoi, Vietnam; 2 MIVEGEC, IRD, CNRS, University of Montpellier, Montpellier, France; 3 Faculty of Animal Science and Veterinary Medicine, Nong Lam University, Ho Chi Minh city, Vietnam; 4 Sub-Department of Animal Health and Production (SDAHP), Cao Lanh, Dong Thap Vietnam; 5 University of Can Tho, Can Tho, Vietnam; 6 Avian Health Research Unit, Chulalongkorn University, Bangkok, Thailand; 7 Food and Agriculture Organization of the United Nations, Jakarta, Indonesia; 8 Nuffield, Department of Medicine, Centre for Tropical Medicine and Global Health, Oxford University, Oxford, UK; 9 Institute of Infection and Global Health, University of Liverpool, Liverpool, UK; 10 LMI “Drug Resistance in South-east Asia” (DRISA), Hanoi, Vietnam

**Keywords:** Antimicrobial usage, Chicken farm, Low- and middle-income country, naïve Bayes model

## Abstract

**Background:**

Antimicrobials are used by poultry farmers in Vietnam as a tool to treat and prevent infectious diseases. We aimed to determine the fraction of disease episodes likely to remain untreated due to the administration of antimicrobials on non-susceptible pathogens in chicken flocks in the Mekong Delta of Vietnam. Weekly data on antimicrobial use and clinical signs were collected from 88 randomly chosen chicken flocks over 124 full production cycles (i.e. time between restocking flocks with day-old chicks and sale for slaughter). A naïve Bayes model was trained to infer the probabilities of disease episodes having been caused by each of 24 pathogens, given the observed clinical sign profile, and expert knowledge on their relative incidence.

**Results:**

A total of 224 disease episodes were observed, of which 44.8% were attributed to viruses (95% CI 31.1–58.4%), 54.6% (CI 40.4–68.7%) to bacteria, and 0.6% (CI 0–1.7%) to a protozoan (*Eimeria* spp.). Antimicrobials were more frequently administered on weeks with disease than on weeks without disease (43.3% vs. 17.8%; *p* < 0.001). A median of 2 [IQR 0–4] antimicrobials were used by episode. The choice of specific antimicrobials was independent on whether the flocks had disease clinical signs or not. Antimicrobials were not used in 30.3% of the episodes. The overall probability that episodes were not effectively treated was 74.2, and 53.7% when discounting cases where the inferred aetiology is viral. Considering only episodes where antimicrobials were given, these probabilities were 57.4 and 23.8% respectively.

**Conclusions:**

This study highlights untargeted use of antimicrobials on small-scale Vietnamese chicken farms, as well as the limitations of antimicrobials as effective tools to control infectious diseases.

**Electronic supplementary material:**

The online version of this article (10.1186/s12917-019-1947-0) contains supplementary material, which is available to authorized users.

## Background

Resistance against antimicrobials (“antimicrobial resistance”, AMR) in animal production has received a great deal of attention in recent times, particularly given its serious implications for human health [1–3]. Zoonotic transmission of resistant organisms or AMR-encoding genes may result from environmental exposure of humans to livestock or its excreta, and/or from the transmission of livestock-borne resistant bacteria/genes through the food chain [4]. Antimicrobials are useful tools to control infectious diseases in animal populations [5]. Recently a consensus has built around the need to restrict their use other than for strict therapeutic purposes, in order to limit the emergence of antimicrobial resistant bacteria [6]. AMR in bacterial pathogens is hypothesized to reduce the effectiveness of antimicrobials in livestock production systems leading to lower levels of profitability and productivity of these systems [7].

With over 100 million tons of meat produced per year (2014), chicken represents the second most common animal food commodity worldwide [8]. Antimicrobials are extensively used in poultry farming, especially in low- and middle-income countries (LMICs) [9]. Faced with an episode of disease in the flock, the administration of antimicrobials is likely to be ineffective when there is mismatch between the chosen antimicrobials and the causative pathogens. This is expected when antimicrobials are administered to treat diseases caused by (1) a non-target organism (i.e. a virus, a fungus, or an intrinsically resistant parasite or bacterial strain), or (2) bacterial organisms that have acquired resistance to the antimicrobial. This is likely to be a common situation where the aetiological agent is not adequately diagnosed, and the choice of antimicrobial is not based on diagnostic or antimicrobial susceptibility testing results. Since veterinary diagnostics are normally not available to small-scale farmers typical of many developing countries, the antimicrobial susceptibility patterns of bacterial organisms is unknown, and choice of antimicrobials is mostly determined by their availability and cost.

Here we develop an original naïve Bayes model approach integrating clinical signs and weekly antimicrobial use (AMU) data from 124 chicken production cycles in 88 small-scale farms of the Mekong delta, Vietnam, allowing to estimate to what extent disease episodes are not effectively treated. Ineffective treatments are expected to fail to treat the disease, leading to a cost due not only to the treatment itself, but also to the loss of production. Ineffective treatments are also likely to contribute to increase the level of resistance in both commensal and pathogenic bacteria. Our method makes full use of available information from the literature and expert opinion in view of the considerable information gaps on diagnostics and antimicrobial sensitivity test (AST), which is often the case in LMICs. These are also the countries that bear the greatest burden of AMR infections [10]. There is unfortunately no way to validate our method. However, since the whole approach is entirely probabilistic, we were able to quantify and accumulate sources of uncertainty along the different steps of the analysis, building confidence intervals around our final estimates. Thus, if not perfect, this method has the advantage of being affordable whilst providing estimates that take into account any uncertainties about the data. Our method may not be useful to improve the situation of a particular farm but it is likely to be of invaluable use in giving recommendations for a local geographical level (district of province).

## Methods

### Farm selection and on-farm data collection

Eighty-eight (88) small-scale farms raising meat chicken flocks were randomly selected from the official census held by the veterinary authorities of Dong Thap province (Mekong Delta, Vietnam) (Sub-Department of Animal Health and Production, SDAHP) in the Cao Lanh and Thap Muoi districts, as part of the “baseline” (observational) phase of a research project [11]. All study farms restocked with 100–2000 chickens for each cycle of production. The chickens are predominantly of native breeds, with a growing period of 3–5 months. The farmers typically sell their birds to local markets with limited household consumption, and their inputs, including day-old-chicks, are also sourced locally. Farmers were provided with a structured diary and were instructed to weekly record information including: (1) clinical signs of disease in the flock: malaise (i.e. prostration, unwillingness to move, ruffled feathers), respiratory distress (sneezing, coughing, nasal/ocular discharge, difficult breathing), diarrhoea (watery faeces), alterations of the central nervous system (CNS) (ataxia, circling, torticollis), leg lesions, sudden death (i.e. death with no clinical signs); and (2) use of antimicrobial drugs (commercial products). Farmers were trained by SDAHP veterinarians on recognition of the six above-listed clinical signs, and were asked to keep containers of all antimicrobial products used. For each production cycle, farms were visited four times, during which records in the farm’s diary were checked, and labels of antimicrobial products used reviewed. Individual antimicrobial active ingredients were entered into a dedicated database through a web application. All visits and data entry were carried out by trained veterinarians affiliated to the SDAHP.

### Expert opinion on disease frequency

Three veterinarians based in Southeast Asia with experience in poultry medicine were independently asked to score the frequencies of 25 common chicken infectious diseases in the region. These pathogens included 14 bacteria: *Listeria monocytog*enes, *Avibacterium paragallinarum*, *Chlamydia psittaci*, *Clostridium perfringens*, *Escherichia coli*, *Erysipelothrix rhusiopathiae*, *Gallibacterium anatis*, *Mycoplasma gallisepticum*, *Ornithobacterium rhinotracheale*, *Pasteurella multocida* (acute and chronic infections), *Pseudomonas* spp., *Salmonella* Gallinarum, *Salmonella* Pullorum, *Staphylococcus aureus*; 9 viruses: Avian Encephalomielitis virus, Highly Pathogenic Avian Influenza (HPAI) virus, Avian Metapneumovirus, Chicken Anaemia virus, Infectious Bursal disease (Gumboro) virus, Infectious Bronchitis virus, Infectious Laryngotracheitis virus, Marek’s disease virus, Newcastle disease virus; and 1 protozoarian parasite (*Eimeria* spp.). The scores of each expert were then scaled to sum up 100, in order to produce values of relative frequency and the average of these 3 scorings was considered in the analysis. Because we distinguished between the acute and chronic infections caused by *Pasteurella multocida*, we will refer to 25 “pathogens” instead of 24 in the rest of the article.

### Aetiology and antimicrobial resistance data from the literature

**Fig. 1:**
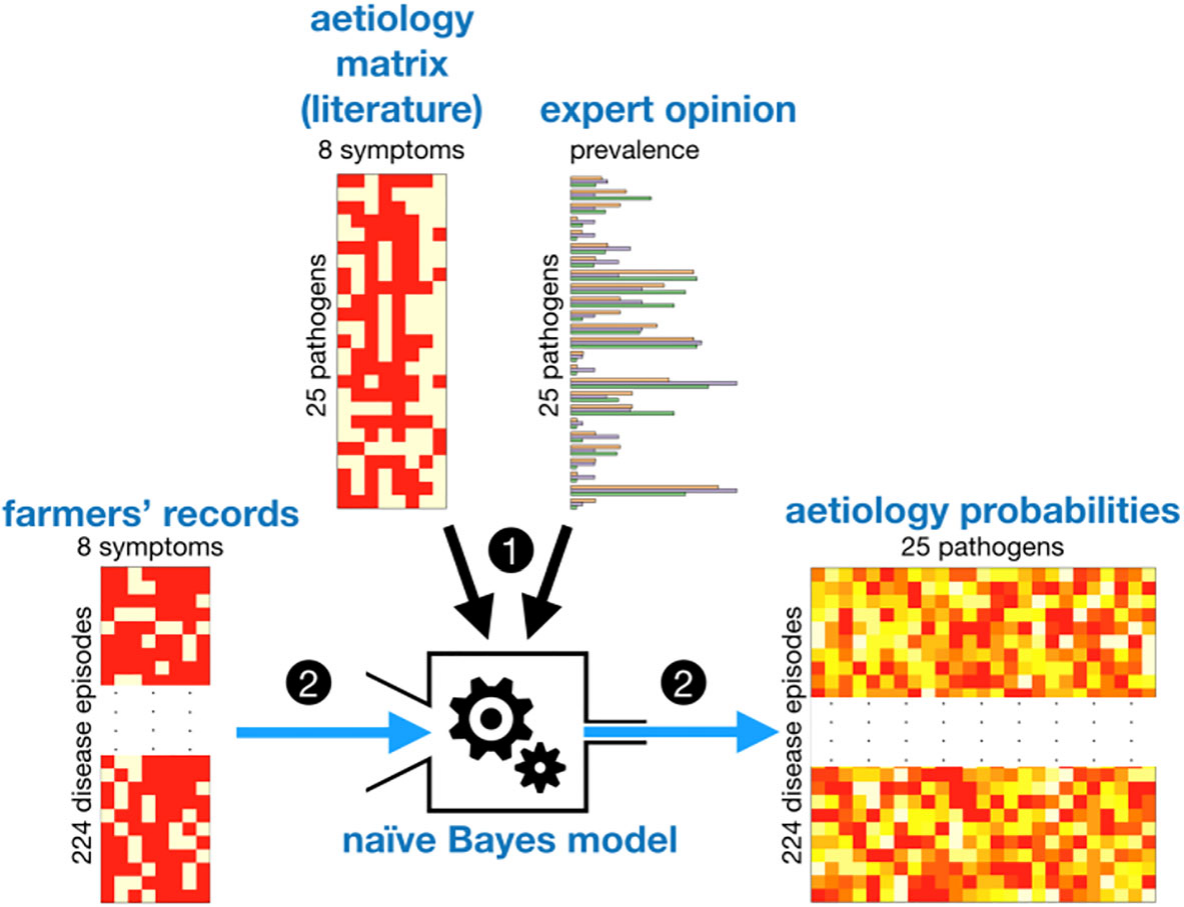
Inferring the aetiologies of diseases episodes. (1) A 25 × 8 presence/absence matrix of clinical signs (and age of infection) per pathogen and the average relative prevalence scores from 3 independent veterinarian experts (top) are used to train a naive Bayes model (centre). (2) The naive Bayes model is then used to convert, for each disease episode, clinical signs and age surveillance data (presence/absence, left) into a vector of aetiology probabilities (right)

We reviewed standard veterinary textbooks on avian diseases [12, 13] to compile a presence/absence matrix of the 6 above-listed clinical signs caused by the 25 above-listed pathogens. We added to this matrix age information, i.e. whether the pathogens are reported for young (< 7 week-old) and old (> 6 week-old) individuals, producing a final “aetiology” matrix of 25 (pathogens) rows and 6 (clinical signs) plus 2 (young and old) columns (Fig. 1).

**Fig. 2:**
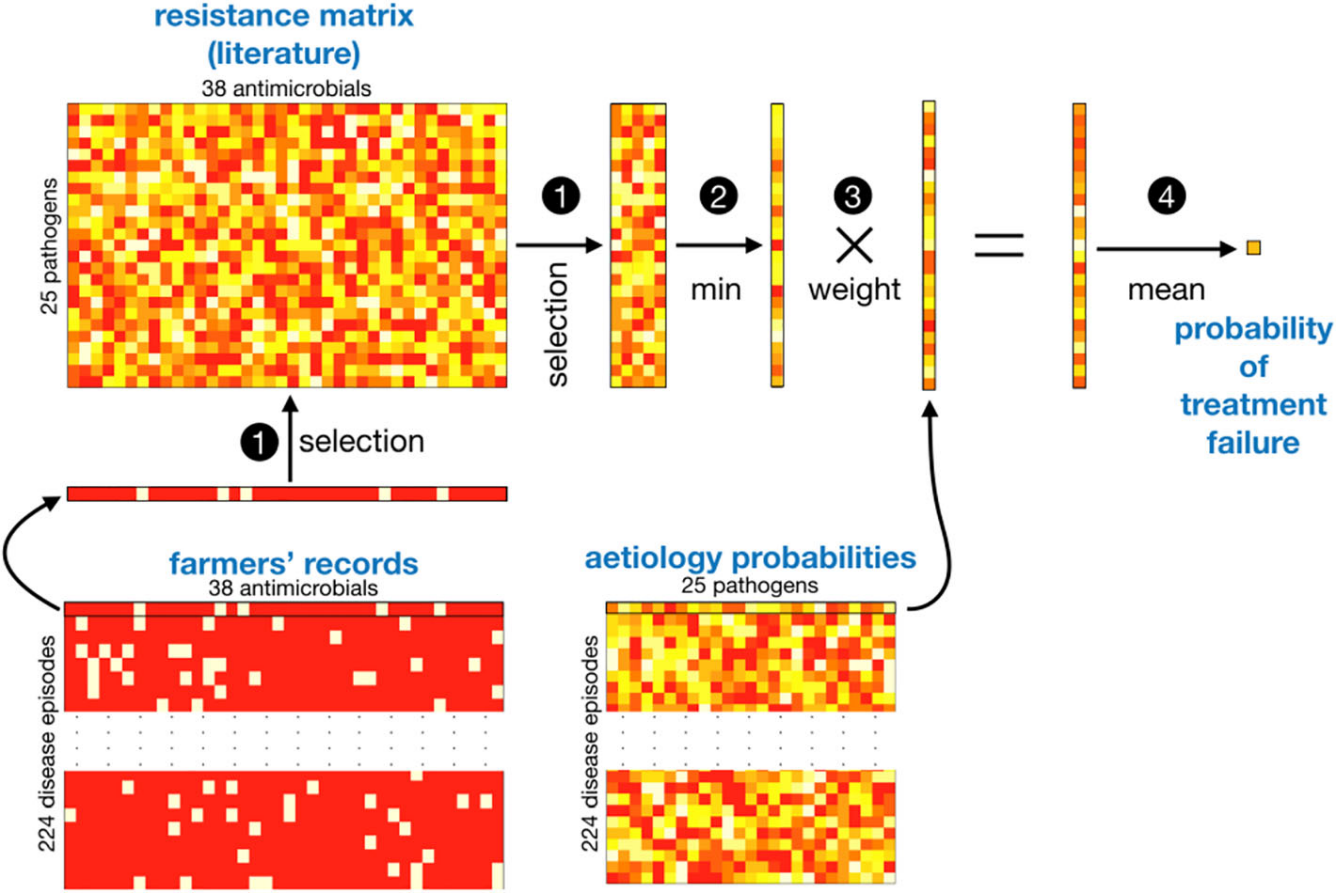
Computing the probability of treatment failure of disease episodes. (1) For each disease episode, we subset the resistance matrix with the drugs used during the disease episode. (2) Then, for each pathogen of the subsetted matrix, we select the minimal level (min) of resistance across the used drugs. (3) The resulting vector is weighted (element-wise multiplication) by the vector of aetiology probabilities computed for that disease episode by the naive Bayes model (Fig. 1); and, (4) The mean of that weighted vector is used as the probability of treatment failure in the disease episode

We used a recently published literature review on the resistance of bacterial pathogens against antimicrobials [14] to produce a “resistance” matrix of 25 (pathogens) rows and *n* (drugs) where *n* was the total number of drugs recorded during the study, see Fig. 2. Each cell of this matrix contains values between 0 (fully susceptible) and 1 (fully resistant), quantifying the resistance of a pathogen to an antimicrobial drug. Missing values from a given drug/pathogen combination were imputed from the mean of the values for the drugs of the same class and the same pathogen when possible. When imputation was not possible (because absence of data on all the drugs of one class), we considered the mean of the values given by the three independent veterinarian experts.

### Analysis

A “disease episode”, defined as a succession of weeks during which clinical signs were reported, was considered out unit of analysis. In order to account for deficiencies in detecting/reporting clinical signs on farm, we assumed that single weeks without clinical signs but preceded and followed by weeks where clinical signs were reported were all part of the same disease episode. A disease episode was then characterized by the set of clinical signs observed and the set of antimicrobials administered during any week of the episode.

The analysis was then developed in two stages. The first one consisted in inferring the aetiologies of disease episodes from their sets of clinical signs, as well as the aetiology matrix and the expert opinion data, using a naive Bayes model framework [15]. The aetiology matrix was used to train the model, and expert opinion data was used as prior information. Note that here, in absence of diagnostic tests, the training phase did not include any validation step. The aetiology matrix from the literature was the only source of information available to train the model. Once trained, the model was applied to the set of clinical signs of each disease episode in order to derive a vector of 25 probabilities (adding up to 1), each probability of that vector quantifying the relative chance that the disease episode was caused by a particular pathogen. We used a Laplace correction factor of 1 in order to account for the fact that observed combinations of clinical signs may not perfectly match any of the combinations of the aetiology matrix. The successive steps leading to the inference of aetiologies of disease episodes are sketched in Fig. 1.

In the second stage of the analysis, for each disease episode, the above-derived aetiology probabilities were then used together with the set of antimicrobials used during the episode and the resistance matrix in order to derive the probability that the applied treatment was ineffective for treating the disease. For that, the resistance matrix was subsetted by column for the drugs used during the disease episode. The minimal values by row (i.e. for a given pathogen) were then computed, producing a vector column of 25 values for the 25 pathogens. The values of this vector were weighted (element-wise multiplications) by the values of the vector of aetiology probabilities and then averaged, producing a probability that the used antimicrobials are ineffective in treating the disease. The successive steps leading to inference of this probability are sketched in Fig. 2.

## Results

### Farms, production cycles and disease episodes

**Fig. 3:**
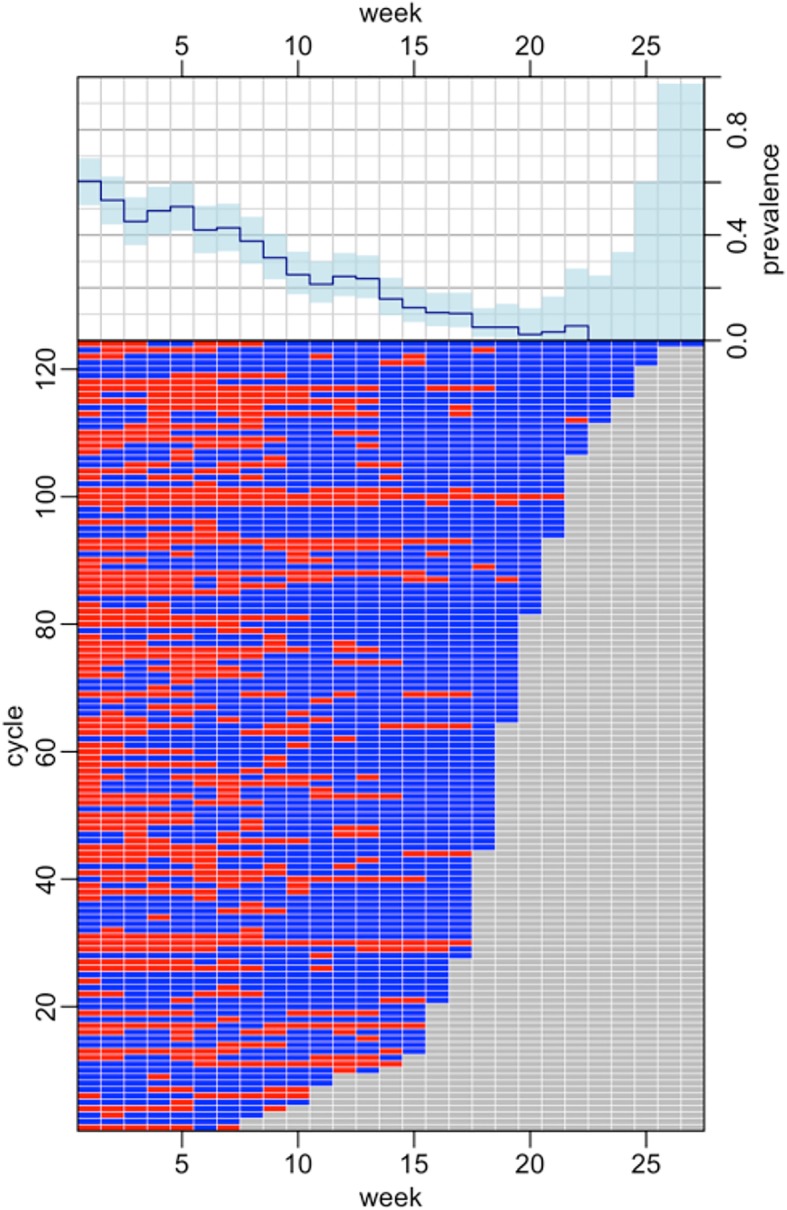
Disease episodes of over the 124 full cycles of production. Top: prevalence of clinical signs of disease on farms (with 95% confidence interval), by week. Bottom: production weeks with (red) and without (blue) disease episode

The 88 farms were followed to include a total of 124 full production cycles (54 over one cycle, 32 over 2 cycles; 2 over 3 consecutive cycles). A total of 224 disease episodes were observed over all cycles. The median duration of one cycle of production was 18 [IQR 17–20] weeks. Clinical signs were recorded in 116/124 (93.5%) cycles of production. The median duration of disease episodes was 2 [IQR 1–4] weeks. Disease episodes spanned a median of 22.7% [IQR 10.0–40.0] observation weeks. The most common clinical signs reported were, in decreasing order, malaise (81.2% episodes), diarrhoea (29.0%), respiratory distress (24.1%), sudden death (15.2%), leg lesions (11.1%), and alteration of the CNS (0.8%). The probability of disease markedly decreased with the age of the flock (Fig. 3).

### Inference of aetiological agents from observed clinical signs

The most common types of clinical signs of the 25 poultry etiologic agents (“aetiology matrix”) are presented in Additional file 1: Table S1. There was reasonable agreement between all three reviewers in their scoring of disease by their relative frequency (r values between 0.78 and 0.89) (Additional file 1: Fig. S1).

Results from the naïve Bayes model expressed as relative probability (by episode and by cycle of production) are presented in Table 1. There was a very strong correlation between the relative probability of each pathogen expressed by week and by episode (r = 0.954; *p* < 0.001). The model attributed 44.8% (95% CI 31.1–58.4%) episodes to viral pathogens, 54.6% (95% CI 40.4–68.7%) to bacterial pathogens, and 0.6% (95%CI 0–1.7%) to *Eimeria* spp. (Table 2). The bacterial infections most commonly predicted were, in decreasing order: (1) *Erysipelothrix rhusiopathiae* (probability per episode 0.079); (2) *Gallibacterium anatis* (0.073); (3) *Mycoplasma gallisepticum* (0.068); (4) *Salmonella* Pullorum (0.068), and *S*. Gallinarum (0.043). The most commonly predicted viral infections were, in decreasing order: (1) Infectious Bursal disease (IBD) (0.162); (2) Avian Metapneumovirus infection (0.105); (3) Marek’s disease (0.057); (4) Infectious Laryngotracheitis (0.038); and (5) Newcastle disease (0.034) (Table 1). There was a strong positive correlation between the probability attributed to a bacterial pathogen and the duration of episodes (r = 0.37; p < 0.001).

**Table 1:** Average probabilities (with 95% confidence intervals) of each of the pathogens (in row) to be the etiological cause of a disease episode or a disease episode in a cycle of production. Note that the probabilities do not necessarily sum to 1 by row because they are averages by episode and cycle of production. Note also that the probabilities averaged by episode can be compared to the mean of the score of the 3 independent experts

Pathogen	Episode			Cycle of production	
	Model	95% CI	Expert opinion	Model	95% CI
Infectious Bursal Disease virus	0.162	0.113–0.210	0.101	0.303	0.101–0.506
Avian Metapneumovirus	0.105	0.064–0.145	0.014	0.194	0.044–0.345
*Erysipelothrix rhusiopathiae*	0.079	0.044–0.115	0.009	0.147	0.044–0.251
*Gallibacterium anatis*	0.073	0.039–0.107	0.008	0.137	0.030–0.244
*Mycoplasma gallisepticum*	0.068	0.035–0.101	0.074	0.127	0.029–0.225
*Salmonella* Pullorum	0.068	0.035–0.101	0.042	0.127	0.042–0.213
Marek’s Disease virus	0.057	0.026–0.087	0.059	0.105	0.000–0.219
*Salmonella* Gallinarum	0.043	0.016–0.069	0.028	0.080	0.028–0.133
Infectious Laringotracheitis virus	0.038	0.013–0.063	0.022	0.036	0.007–0.064
*Clostridium perfringens* (necrotic) enteritis)	0.038	0.013–0.063	0.059	0.071	0.004–0.138
*Escherichia coli* (colibacillosis)	0.034	0.011–0.058	0.106	0.063	0.023–0.102
Newcastle Disease virus	0.034	0.010–0.057	0.079	0.064	0.000–0.133
*Chlamydia psittaci*	0.034	0.010–0.057	0.006	0.063	0.016–0.111
*Staphylococcus aureus*	0.032	0.009–0.054	0.024	0.059	0.013–0.105
Chicken Anaemia virus	0.031	0.008–0.053	0.022	0.057	0.016–0.098
*Pasteurella multocida* (acute) (fowlcholera)	0.025	0.004–0.045	0.035	0.047	0.003–0.091
*Ornithobacterium rhinotracheale*	0.023	0.003–0.042	0.025	0.042	0.007–0.077
*Listeria monocytogenes*	0.012	0.000–0.026	0.009	0.022	0.000–0.043
Infectious Bronchitis virus	0.011	0.000–0.024	0.060	0.020	0.008–0.033
Avian Encephalomielitis virus	0.009	0.000–0.021	0.011	0.008	0.000–0.016
*Avibacterium paragallinarum*	0.008	0.000–0.020	0.031	0.015	0.000–0.032
*Eimeria* spp.	0.006	0.000–0.017	0.038	0.012	0.003–0.022
*Pasteurella multocida* (chronic)	0.005	0.000–0.015	0.011	0.010	0.000–0.020
*Pseudomonas* spp.	0.005	0.000–0.014	0.011	0.009	0.000–0.018
Highly Pathogenic Avian Influenza virus	0.002	0.000–0.008	0.113	0.004	0.000–0.011

**Table 2:** Average probabilities (with 95% confidence intervals) that a disease episode caused by a given bacteria (by row) remains untreated either because of absence of treatment or because of ineffective treatment (first two columns), or because of ineffective treatment only (last two columns). The probabilities in the last two columns are necessarily smaller than in the first two columns

Bacterial pathogen	Overall treatment failure		Treatment ineffective when antimicrobial/s given	
	Mean	95% CI	Mean	95% CI
*Avibacterium paragallinarum*	0.499	0.433–0.564	0.175	0.125–0.224
*Chlamydia psittaci*	0.554	0.488–0.619	0.265	0.207–0.322
*Clostridium perfringens* (necrotic enteritis)	0.595	0.530–0.659	0.332	0.271–0.394
*Eimeria* spp.	0.895	0.855–0.935	0.827	0.778–0.877
*Erysipelothrix rhusiopathiae*	0.611	0.547–0.675	0.360	0.297–0.422
*Escherichia coli* (colibacillosis)	0.555	0.490–0.620	0.266	0.209–0.324
*Gallibacterium anatis*	0.562	0.497–0.627	0.278	0.220–0.337
*Listeria monocytogenes*	0.602	0.538–0.666	0.344	0.282–0.406
*Mycoplasma gallisepticum*	0.645	0.582–0.707	0.415	0.350–0.479
*Ornithobacterium rhinotracheale*	0.650	0.587–0.712	0.423	0.359–0.488
*Pasteurella multocida* (*acute* (fowl cholera))	0.411	0.347–0.475	0.030	0.008–0.052
*Pasteurella multocida* (chronic)	0.411	0.347–0.475	0.030	0.008–0.052
*Pseudomonas* spp.	0.479	0.413–0.544	0.142	0.096–0.187
*Salmonella* Gallinarum	0.425	0.360–0.490	0.053	0.024–0.082
*Salmonella* Pullorum	0.425	0.360–0.490	0.053	0.024–0.082
*Staphylococcus aureus*	0.472	0.407–0.537	0.130	0.086–0.175
Overall (all episodes)	0.537	0.472–0.603	0.238	0.182–0.294

**Fig. 4:**
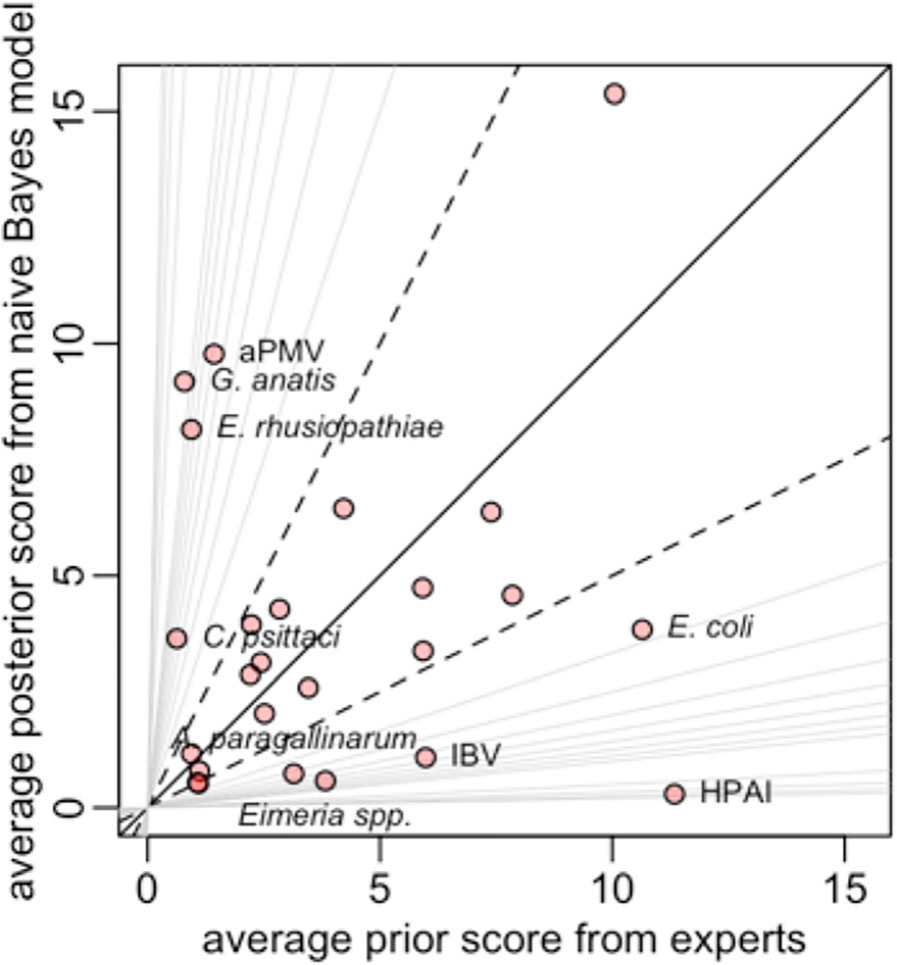
Relationship between prior estimates from veterinary expert opinion and posterior probabilities predicted by the naïve Bayes model. Lines above the diagonal have slopes increasing from 2 (black dashed line), 3, … 10, 20, …50 (all grey lines). Similarly, lines below the diagonal have slopes decreasing from ½ (black dashed line), 1/3, … 1/10, 1/20, … 1/50 (all grey lines)

Overall, there was a reasonable agreement between the prior probabilities estimated by the naïve Bayes model and the average of the three poultry veterinary experts. However, the assessments of the experts on HPAI, *E. coli*, Infectious Bronchitis virus, *Avibacterium paragallinarum* and *Eimeria* spp. are higher than the incidences predicted by the model (Fig. 4). Conversely, their assessments on Avian Metapneumovirus, *G. anatis*, *E. rhusiopathiae*, and *Clamydia psittaci* are lower than the incidences predicted by the model (Fig. 4).

### Antimicrobial use and disease episodes

**Fig. 5:**
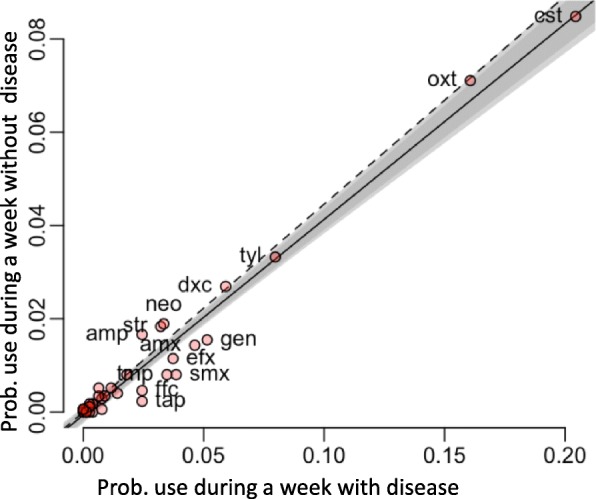
Probability of use of antimicrobial active ingredients in weeks with and without disease. Relationship, for each antimicrobial, between the probability of use during a week with disease and without disease. The black line is the regression line and the light and grey areas are the 99 and 95% confidence intervals respectively. The dashed line is the expected relationship in case there is no difference of usage between the weeks with and without disease (the slope is equal to the ratio of weeks with and without disease). cst = colistin, oxt = oxytetracycline, tyl = tylosin, dxc = doxycycline, neo = neomycin, gen = gentamicin, str = streptomycin, amp = ampicillin, amx = amoxicillin, gen = gentamicin, efx = enrofloxacin, tmp = trimethoprim, smx = sulfamethoxazole, ffc = florfenicol, tmp = thiamphenicol

Antimicrobials were more frequently administered on disease episode weeks (296/683, 43.3%), than in weeks without disease (281/1582, 17.8%) (χ^2^ = 163.0, *p* = 0.001). Similar to the probability of disease, the weekly probability of antimicrobial usage decreased with the age of the flock: from 0.84 (week 1), to 0.31–0.44 (weeks 2–7), 0.10–0.30 (weeks 8–15), and < 0.10 thereafter. Farmers did not use antimicrobials in 88/224 (39.3%) of disease episodes. Thirty-eight (38) different types of antimicrobials were used by farmers (Additional file 1: Table S2). The most frequently used antimicrobials were: colistin (12.2% weeks across farms), oxytetraycline (9.8%), tylosin (4.8%), and doxycycline (3.7%). These four antimicrobials represented 53.1% of total usage. In episodes where antimicrobials were used, the median number of different antimicrobials used was 3 [IQR 2–4]. There was no evidence that different antimicrobials are more likely to be used in situations of disease, compared with no disease (Fig. 5). Episodes where no antimicrobials were used had a shorter duration (median 1 [IQR 1–2] weeks) compared with episodes where antimicrobials were used (median 3 [IQR 1–5] weeks) (Wilcoxon test, W = 3120; *p* < 0.001).

### Phenotypic resistance of bacterial organisms

The full list of antimicrobials used, alongside the prevalence of resistance of poultry pathogens against them is presented in Additional file 1: Table S2.

### Probability that disease in flocks remains untreated

**Fig. 6:**
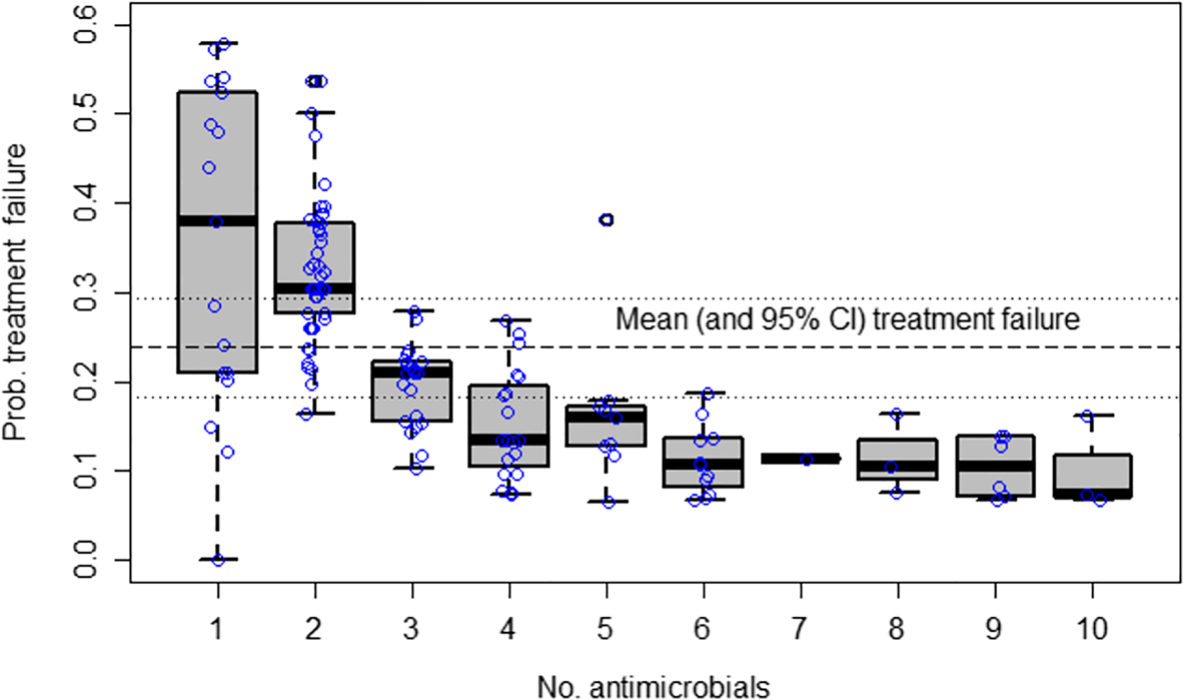
Predicted summary treatment failure of individual episodes attributed to bacterial pathogens. The box indicates median values and 75% interquartile-range; whiskers indicate extreme values

The overall probability (all episodes) that a disease episode remains untreated (either because of absence of treatment, or because of ineffective treatment) was 74.2% (95% CI 68.4–79.9%) for all episodes, and 53.7% (95% CI 47.2–60.3%) for episodes due to bacterial pathogens (including *Eimeria* spp.). For episodes where antimicrobials were given, the estimated treatment failure was 57.4 (51.0–63.9%) (all pathogens), and 23.8% (95% CI 18.2–29.4%) (bacterial pathogens). The probability of failing to treat the disease in episodes where antimicrobials were given was very variable, ranging from 0.423 (*Ornithobacterium rhinotracheale*) to 0.030 (*Pasteurella multocida*) (Table 2). For bacterial pathogen, this probability was strongly dependent on the number of antimicrobials used (Fig. 6).

## Discussion

Antimicrobials are formidable tools for the control of infectious diseases in animal production. The trade-offs of antimicrobial usage have been discussed, although focused on their costs vs. the benefits from protecting flocks/herds from disease [16]. This study is, to our knowledge, the first one to look into the likelihood of unsuccessful treatment of infectious diseases in small-scale farming systems in Asia, either because antimicrobials were not used, or because an ineffective antimicrobial were used. Key findings of this study are: (1) half (48.7%) antimicrobial use occurred on weeks without disease; (2) for episodes where antimicrobials were used, they were expected to be ineffective in 57.4% (CI 51.0–63.9%) episodes (for all pathogens considered), and 23.8% (18.2–29.4%) (for bacterial pathogens); (3) antimicrobials were not used in over a third (39%) of disease episodes.

Our analysis estimated that approximately a fourth (23.8%) of treated bacterial episodes are likely to be ineffective due to the organisms treated being resistant to the antimicrobials used. This outcome is likely a combination of “intrinsic” and “acquired” resistance properties of bacterial pathogens. However, in this paper we have not attempted to investigate the fraction likely due to acquired resistance since for many antimicrobials and pathogens this is now well known. Most published AMR data on poultry pathogens comes from studies in developed countries. Given the higher levels of antimicrobial use in Vietnamese chicken farms [17], it is likely that the resulting values of expected antimicrobial resistance are underestimated. We ignored the timing of application of the antimicrobial in relation to the onset of disease, or the order of the administration because this could not be determined from weekly data collection. Surprisingly however, in over a third of disease episodes (39%) farmers gave no antimicrobials at all, resulting in an even higher percent in overall failure to efficiently treat a bacterial disease episode (53.7%). When viruses are also considered, the overall fraction of treatment failure reached 74.2%, as ~ 45% of disease episodes were expected to be caused by viral pathogens.

Two assumptions of our study may have resulted in biased results. Firstly, the assumption that all disease episodes were either due to a bacterial or a viral pathogen, excluding helminth infections and other non-infectious aetiologies (i.e. toxicosis, metabolic disorders, etc.). However, given the farming conditions of small-scale farms in Vietnam, with generally serious deficiencies in biosecurity, it is likely that the overwhelming majority of over disease is infectious in nature. Secondly, the study is necessarily biased towards diseases that are easier to diagnose/detect. Interestingly the expert panel predicted HPAI and colibacillosis (*E. coli*) to be more common than what the model predicted after integrating data on clinical signs. Further diagnostic testing in the area by the authors has confirmed a lack of HPAI in the areas at the time of the study (data not shown). Surprisingly, the model and the experts predicted generally relatively low incidence of coccidiosis (*Eimeria* spp.), which is regarded as a major health problem in industrialised poultry production systems. It is believed that coccidial infectious are indeed present, but mostly the subclinical form is predominant, contributing to reduced intestinal functions [18]. Thirdly, we ignored data on vaccination (mostly to prevent viral infections) and assumed that the probability of an episode due to a given virus was not affected by whether the flock had been vaccinated or not. Farmers in the area apply vaccines notably against HPAI, IBD and Newcastle disease. However, the application of the vaccine requires careful logistics including adequate strain choice and logistics (timing, booster, storage and administration logistics) than more often than not were not met. In the case of HPAI, there is some evidence that vaccination coverage is either low or application is performed poorly [19].

A third of disease episodes did not trigger farmers to administer antimicrobials. These episodes were typically short (one week) with non-specific signs of disease (i.e. malaise). Often in these cases, farmers used vitamins, probiotics, yeasts and antibodies to manage poultry health issues (data not shown). Interestingly, episodes attributed to bacteria tended to last longer, giving further empirical evidence to the phenomenon of AMR on farms.

Although most episodes were addressed by the administration of two antimicrobials, in some instances up to 10 different antimicrobial active principles were used by the farmer. This is not surprising, since many commercial antimicrobial formulations in the area include at least two antimicrobial active ingredients [20] and confirms high usage of antimicrobials in Vietnamese small-scale chicken farms [20, 21]. However, over 50% of total antimicrobial use corresponded to weeks with no disease reported (i.e. prophylactic use). This is likely to be partly triggered by fear of disease, either from previous experience or by the knowledge of presence of nearby disease, coupled with the lack of competent veterinary diagnostic/advisory capacity. As suggested in the introduction, there is a strong suspicion that the choice of antimicrobials is currently based on costs.

Some of the most commonly used antimicrobials (i.e. colistin, oxytetracycline) were associated with a high probability of ineffective treatment of the disease (data not shown). In the case of colistin, this reflects a high predicted incidence of *Gallibacterium anatis* infection (characterized by respiratory, diarrhoea and malaise, in all ages), and *Erysipelothrix rhusiopathiae* (malaise, sudden death, in all ages), both of which are often very resistant against these antimicrobials (≥40%). To the best of our knowledge, *Gallibacterium anatis* has never been isolated in Vietnam. Our results suggest that it could be valuable to include this pathogen in the diagnostic testing protocols. The use of colistin (and to lesser extent fluoroquinolones, macrolides, aminoglycosides and β-lactams), some of which are considered of critical importance for human medicine [22] is particularly worrying from a public health point of view.

Our approach is particularly useful in settings where diagnostic capacity (and AMR testing) is limited, such as many LMICs [23]. As more local epidemiological and microbiological data becomes available, through improved diagnostic and AMR testing, these can easily be integrated in our modelling framework to improve the precision and accuracy of our estimates. The approach can also help to focus diagnostic efforts towards those diseases that are considered more likely, as well as to review vaccination programmes. In generally, the model framework we developed here can be used for any system (animal or human) where clinical signs, antimicrobial use and AMR data are known to improve treatment success.

In summary, using a novel integrated methodology that combined data from expert opinion, literature and field observations, we investigated the relationship between AMU and infectious disease in smallholder poultry systems. When farmers used antimicrobials to address disease episodes in their flocks, failure to treat disease was expected in about ~ 57% cases (~ 24% assuming a bacterial causative agent). Our study shows a high frequency of usage of antimicrobials in situations with no disease, and absence of use when disease is present on flocks, the widespread use of multiple courses of different antimicrobials, and the random use of different antimicrobial products suggesting that there is ample room for improvement in the targeting of antimicrobials on farms in small-scale farming systems in Vietnam.

## Conclusions

This study shows how clinical signs and antimicrobials usage surveillance data can be used to infer the level of antimicrobial misuse in chicken farms. The naïve Bayes framework that we employ allows to do so probabilistically, rigorously accounting for all sources of uncertainty. Our results show that a vast majority of disease episodes are likely to be not treated effectively, representing an important loss for the farmers. The method that we develop is general and can be applied to any set-up, including human infections. The model can also be used to improve the current treatments at use.

## Additional file

Additional file 1: **Figure S1:** relationships and correlations between the scores of the 3 independent veterinarian experts on the frequencies of the 25 pathogens. **Table S1:** presence/absence aetiology matrix. “X” are used whenever a symptom (in column) has been reported for a given infection (in row) in standard veterinary textbooks on avian diseases (9, 10). **Table S2** Prevalence of resistance (in percentages) of the 16 poultry pathogens against the 39 antimicrobials considered in the study. Values come either from the literature (blue), expert opinion (green), or are inferred from the values of other antimicrobials in the same class (yellow). (DOCX 106 kb)

## Data Availability

All the data sets used in this study as well as R code are available from 10.5281/zenodo.2611133, or https://github.com/viparc/treatfail for an up-to-date version.

## Abbreviations

AMR: Antimicrobial resistance
AMU: Antimicrobial use
AST: Antimicrobial sensitivity test
CI: Confidence interval
CNS: Central nervous system
HPAI: Highly pathogenic avian influenza
IBD: Infectious bursal disease
IQR: Interquartile range
LMIC: Low- and middle-income countries
OXTREC: Oxford tropical research ethics committee
SDAHP: Sub-department of animal health and production
