# Supplementary material for: Assessing antimicrobial misuse in small-scale chicken farms in Vietnam from an observational study
Source: BMC Vet Res. 2019 Jun 20;15:206. doi: 10.1186/s12917-019-1947-0 (PMC6585117; doi:10.1186/s12917-019-1947-0)
Supplement: Supplementary file 1 — Figure S1: relationships and correlations between the scores of the 3 independent veterinarian experts on the frequencies of the 25 pathogens. Table S1: presence/absence aetiology matrix. “X” are used whenever a symptom (in column) has been reported for a given infection (in row) in standard veterinary textbooks on avian diseases (9, 10). Table S2 Prevalence of resistance (in percentages) of the 16 poultry pathogens against the 39 antimicrobials considered in the study. Values come either from the literature (blue), expert opinion (green), or are inferred from the values of other antimicrobials in the same class (yellow). (DOCX 106 kb) [file 12917_2019_1947_MOESM1_ESM.docx]

**Additional file 1**

**
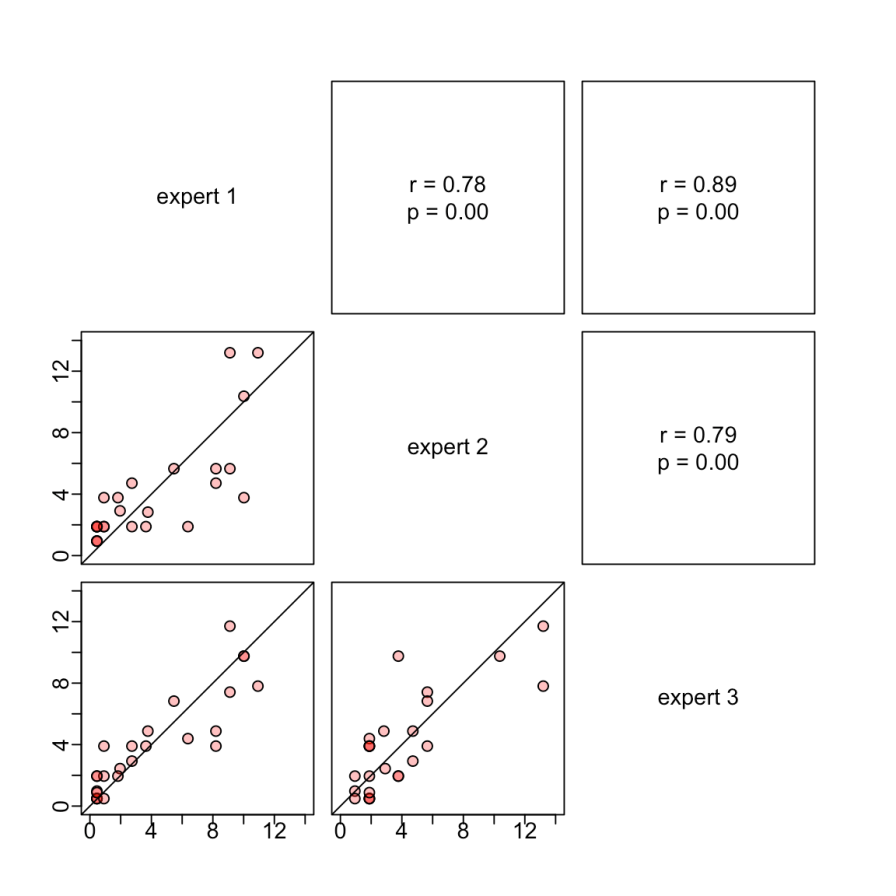
**

**Figure S1:** relationships and correlations between the scores of the 3 independent veterinarian experts on the frequencies of the 25 pathogens.

**Table S1:** presence/absence aetiology matrix. “X” are used whenever a symptom (in column) has been reported for a given infection (in row) in standard veterinary textbooks on avian diseases (9, 10).

| pathogen | young birds | old birds | respiratory | diarrhoea | CNS | malaise | leg lesions | sudden death |
| --- | --- | --- | --- | --- | --- | --- | --- | --- |
| *Pasteurella multocida* (fowl cholera) |  | X | X | X |  | X |  | X |
| *Pasteurella multocida* (chronic) |  | X |  |  |  | X | X | X |
| *Erysipelothrix rhusiopathiae* | X | X |  | X |  | X |  | X |
| *Chlamydia psittaci* | X | X | X | X |  | X |  |  |
| *Pseudomonas* spp. | X |  | X | X |  |  |  | X |
| *Ornithobacterium rhinotracheale* | X |  | X |  |  |  |  |  |
| *Avibacterium paragallinarum* |  | X | X |  |  |  |  |  |
| *Gallibacterium anatis* | X | X | X | X |  | X |  | X |
| *Mycoplasma gallisepticum* | X | X | X |  |  |  |  |  |
| *Clostridium perfringens* (Necrotic enteritis) |  | X |  | X |  | X |  |  |
| *Escherichia coli* (colibacillosis) | X |  | X |  |  | X |  | X |
| *Salmonella* *pullorum* | X |  |  | X |  | X |  |  |
| *Salmonella* *gallinarum* | X | X |  | X |  | X |  |  |
| *Staphylooccus aureus* | X | X |  |  |  | X | X |  |
| Infectious bronchitis | X | X | X |  |  |  |  |  |
| Avian influenza | X | X | X | X | X |  | X | X |
| Gumboro disease |  | X |  | X |  | X |  |  |
| Infectious laringotracheitis | X | X | X |  |  |  |  | X |
| Newcastle disease | X | X |  | X | X | X |  | X |
| Avian metapneumovirus | X | X | X |  |  |  |  |  |
| Avian listeriosis | X | X |  |  | X |  |  | X |
| Marek's disease |  | X |  |  | X |  |  |  |
| Avian encephalomielitis | X |  |  |  | X |  |  |  |
| Chicken anaemia virus | X | X |  |  |  | X |  |  |
| *Eimeria* spp. (coccidiosis) | X |  |  |  |  |  |  |  |

**Table S2** Prevalence of resistance (in percentages) of the 16 poultry pathogens against the 39 antimicrobials considered in the study. Values come either from the literature (blue), expert opinion (green), or are inferred from the values of other antimicrobials in the same class (yellow).

|  | Literature review |
| --- | --- |
|  | Inferred from other antimicrobials in the same class |
|  | Expert opinion |

| Antimicrobial class | Antimicrobial | *Escherichia coli* (colibacillosis) | *Salmonella pullorum* | *Salmonella gallinarum* | *Pasteurella multocida* (acute) (fowl cholera) | *Pasteurella multocida* (chronic pasteurellosis) | *Ornithobacterium rhinotracheale* | *Avibacterium paragallinarum* | *Gallibacterium anatis* | *Clostridium perfringens* (Necrotic enteritis) | *Staphylococcus aureaus* | *Mycoplasma gallisepticum* | *Listeria monocytogenes* | *Erysipelothrix rhusiopathiae* | *Chlamydia psittaci* | *Pseudomonas* spp. | *Eimeria* spp. (coccidiosis) |
| --- | --- | --- | --- | --- | --- | --- | --- | --- | --- | --- | --- | --- | --- | --- | --- | --- | --- |
| Penicillins | AMOXCILLIN | 80.0 | 24.8 | 24.8 | 5.0 | 5.0 | 51.6 | 14.0 | 36.0 | 3.5 | 45.0 | 100.0 | 20.0 | 20.0 | 100.0 | 15.0 | 100.0 |
|  | AMPICILLIN | 82.0 | 13.0 | 13.0 | 2.3 | 2.3 | 40.0 | 38.9 | 36.0 | 0.0 | 45.0 | 100.0 | 20.0 | 20.0 | 100.0 | 15.0 | 100.0 |
| First generation cephalosporins | CEPHALEXIN | 50.0 | 0.0 | 0.0 | 1.8 | 1.8 | 63.0 | 16.8 | 3.0 | 0.0 | 25.0 | 100.0 | 10.0 | 10.0 | 100.0 | 15.0 | 100.0 |
| Third generation cephalosporins | CEFOTAXIME | 28.6 | 0.0 | 0.0 | 1.8 | 1.8 | 63.0 | 16.8 | 3.0 | 0.0 | 15.0 | 100.0 | 10.0 | 10.0 | 100.0 | 15.0 | 100.0 |
| Aminoglycosides | APRAMYCIN | 56.1 | 19.6 | 19.6 | 15.0 | 15.0 | 51.1 | 56.9 | 32.3 | 98.8 | 7.5 | 100.0 | 30.0 | 100.0 | 80.0 | 15.0 | 100.0 |
|  | GENTAMICIN | 30.9 | 2.6 | 2.6 | 4.3 | 4.3 | 93.1 | 46.8 | 4.0 | 100.0 | 15.0 | 100.0 | 30.0 | 100.0 | 80.0 | 15.0 | 100.0 |
|  | SPECTINOMYCIN | 70.1 | 45.0 | 45.0 | 1.2 | 1.2 | 9.1 | 30.5 | 90.0 | 98.8 | 0.0 | 100.0 | 30.0 | 100.0 | 80.0 | 15.0 | 100.0 |
|  | STREPTOMYCIN | 69.0 | 27.0 | 27.0 | 8.5 | 8.5 | 51.1 | 72.7 | 21.0 | 100.0 | 7.5 | 100.0 | 30.0 | 100.0 | 80.0 | 15.0 | 100.0 |
|  | NEOMYCIN | 54.4 | 3.9 | 3.9 | 8.5 | 8.5 | 51.1 | 77.4 | 14.0 | 96.5 | 7.5 | 100.0 | 30.0 | 100.0 | 80.0 | 15.0 | 100.0 |
| Macrolides | ERYTHROMYCIN | 90.0 | 100.0 | 100.0 | 18.0 | 18.0 | 33.8 | 77.8 | 43.0 | 17.5 | 3.8 | 80.0 | 100.0 | 40.0 | 20.0 | 100.0 | 100.0 |
|  | TYLOSIN | 100.0 | 100.0 | 100.0 | 97.0 | 97.0 | 98.0 | 6.0 | 100.0 | 0.0 | 80.0 | 80.0 | 100.0 | 40.0 | 20.0 | 100.0 | 100.0 |
|  | LINCOMYCIN | 94.0 | 100.0 | 100.0 | 51.2 | 51.2 | 85.0 | 90.8 | 71.5 | 62.4 | 8.8 | 80.0 | 100.0 | 40.0 | 20.0 | 100.0 | 100.0 |
|  | SPIRAMYCIN | 94.7 | 100.0 | 100.0 | 55.4 | 55.4 | 96.0 | 58.2 | 71.5 | 26.6 | 4.2 | 80.0 | 100.0 | 40.0 | 20.0 | 100.0 | 100.0 |
|  | JOSAMYCIN | 94.7 | 100.0 | 100.0 | 55.4 | 55.4 | 78.2 | 58.2 | 71.5 | 26.6 | 4.2 | 80.0 | 100.0 | 40.0 | 20.0 | 100.0 | 100.0 |
|  | TILMICOSIN | 99.0 | 100.0 | 100.0 | 2.5 | 2.5 | 54.4 | 58.2 | 71.5 | 26.6 | 4.2 | 80.0 | 100.0 | 40.0 | 20.0 | 100.0 | 100.0 |
| Phenicols | CHLORAMPHENICOL | 63.5 | 0.0 | 0.0 | 3.3 | 3.3 | 25.0 | 0.0 | 2.0 | 23.0 | 5.0 | 20.0 | 60.0 | 60.0 | 60.0 | 50.0 | 100.0 |
|  | FLORFENICOL | 20.9 | 0.0 | 0.0 | 0.6 | 0.6 | 25.0 | 0.0 | 2.0 | 0.0 | 5.0 | 20.0 | 60.0 | 60.0 | 60.0 | 50.0 | 100.0 |
|  | THIAMPHENICOL | 42.2 | 0.0 | 0.0 | 2.0 | 2.0 | 25.0 | 0.0 | 2.0 | 11.5 | 5.0 | 20.0 | 60.0 | 60.0 | 60.0 | 50.0 | 100.0 |
| Tetracyclines | TETRACYCLINE | 91.0 | 11.2 | 11.2 | 13.8 | 13.8 | 21.0 | 80.0 | 90.0 | 66.6 | 47.5 | 40.0 | 60.0 | 40.0 | 20.0 | 90.0 | 100.0 |
|  | CHLORTETRACYCLINE | 94.5 | 5.6 | 5.6 | 11.2 | 11.2 | 38 | 70.2 | 88.0 | 76.8 | 47.5 | 40.0 | 60.0 | 40.0 | 20.0 | 90.0 | 100.0 |
|  | OXYTETRACYCLINE | 98.0 | 5.6 | 5.6 | 8.6 | 8.6 | 38.0 | 66.6 | 94.0 | 65.9 | 47.5 | 40.0 | 60.0 | 40.0 | 20.0 | 90.0 | 100.0 |
|  | DOXYCYCLINE | 95.0 | 0.0 | 0.0 | 11.1 | 11.1 | 55.0 | 63.9 | 80.0 | 98.0 | 47.5 | 40.0 | 60.0 | 40.0 | 20.0 | 90.0 | 100.0 |
| Sulphonamides | SULFACHLOROPYRIDAZINE | 92.5 | 44.5 | 44.5 | 9.0 | 9.0 | 40 | 60.0 | 43.0 | 50.0 | 0.0 | 100.0 | 20.0 | 100.0 | 50.0 | 50.0 | 25.0 |
|  | SULFADIMETHOXINE | 92.5 | 44.5 | 44.5 | 9.0 | 9.0 | 99.2 | 60.0 | 43.0 | 50.0 | 0.0 | 100.0 | 20.0 | 100.0 | 50.0 | 50.0 | 25.0 |
|  | SULFADIMIDINE | 92.5 | 44.5 | 44.5 | 9.0 | 9.0 | 69.6 | 60.0 | 43.0 | 50.0 | 0.0 | 100.0 | 20.0 | 100.0 | 50.0 | 50.0 | 25.0 |
|  | SULFAGUANIDIN | 92.5 | 44.5 | 44.5 | 9.0 | 9.0 | 69.6 | 60.0 | 43.0 | 50.0 | 0.0 | 100.0 | 20.0 | 100.0 | 50.0 | 50.0 | 25.0 |
|  | SULFAMETHOXYPYRIDAZINE | 92.5 | 44.5 | 44.5 | 9.0 | 9.0 | 69.6 | 60.0 | 43.0 | 50.0 | 0.0 | 100.0 | 20.0 | 100.0 | 50.0 | 50.0 | 25.0 |
|  | SULFADIAZINE | 92.5 | 44.5 | 44.5 | 9.0 | 9.0 | 69.6 | 60.0 | 43.0 | 50.0 | 0.0 | 100.0 | 20.0 | 100.0 | 50.0 | 50.0 | 25.0 |
|  | SULFAMETHAZINE | 92.5 | 44.5 | 44.5 | 9.0 | 9.0 | 69.6 | 60.0 | 43.0 | 50.0 | 0.0 | 100.0 | 20.0 | 100.0 | 50.0 | 50.0 | 25.0 |
|  | SULFAMETHOXAZOLE | 92.5 | 44.5 | 44.5 | 9.0 | 9.0 | 69.6 | 60.0 | 43.0 | 50.0 | 0.0 | 100.0 | 20.0 | 100.0 | 50.0 | 50.0 | 25.0 |
|  | SULFATHIAZOLE | 92.5 | 44.5 | 44.5 | 9.0 | 9.0 | 69.6 | 60.0 | 43.0 | 50.0 | 0.0 | 100.0 | 20.0 | 100.0 | 50.0 | 50.0 | 25.0 |
| Pyrimidine inhibitor | TRIMETHOPRIM | 46.5 | 82.8 | 82.8 | 19.5 | 19.5 | 89.0 | 60.0 | 25.0 | 50.0 | 1.3 | 100.0 | 20.0 | 30.0 | 50.0 | 50.0 | 100.0 |
| Quinolones | CIPROFLOXACIN | 67.0 | 2.2 | 2.2 | 4.5 | 4.5 | 82.1 | 33.4 | 4.0 | 58.0 | 3.8.0 | 20.0 | 30.0 | 30.0 | 20.0 | 30.0 | 100.0 |
|  | ENROFLOXACIN | 32.0 | 2.8 | 2.8 | 4.7 | 4.7 | 70.8 | 29.7 | 4.0 | 41.0 | 3.8.0 | 20.0 | 30.0 | 30.0 | 20.0 | 30.0 | 100.0 |
|  | FLUMEQUINE | 80.0 | 1.7 | 1.7 | 40.0 | 40.0 | 93.3 | 31.5 | 4.0 | 55.3 | 3.8.0 | 20.0 | 30.0 | 30.0 | 20.0 | 30.0 | 100.0 |
|  | NORFLOXACIN | 54.0 | 0.0 | 0.0 | 8.1 | 8.1 | 82.1 | 31.5 | 4.0 | 67.0 | 3.8.0 | 20.0 | 30.0 | 30.0 | 20.0 | 30.0 | 100.0 |
|  | OXOLINIC ACID | 58.3 | 1.7 | 1.7 | 14.3 | 14.3 | 82.1 | 31.5 | 4.0 | 55.3 | 3.8.0 | 20.0 | 30.0 | 30.0 | 20.0 | 30.0 | 100.0 |
| Polypeptides | ENRAMYCIN | 100.0 | 100.0 | 100.0 | 100.0 | 100.0 | 100.0 | 100.0 | 100.0 | 5.0 | 100.0 | 100.0 | 100.0 | 25.0 | 100.0 | 100.0 | 100.0 |
| Polymixins | COLISTIN | 12.0 | 0.0 | 0.0 | 0.0 | 0.0 | 100.0 | 20.0 | 100.0 | 100.0 | 100.0 | 100.0 | 100.0 | 100.0 | 100.0 | 0.0 | 100.0 |
